# Short- and long-term outcomes for transvaginal specimen extraction versus minilaparotomy after robotic anterior resection for colorectal cancer: a mono-institution retrospective study

**DOI:** 10.1186/s12957-020-01967-9

**Published:** 2020-07-29

**Authors:** Gengmei Gao, Lan Chen, Rui Luo, Bo Tang, Taiyuan Li

**Affiliations:** 1 Medical College of Nanchang University, Nanchang, 330000 China; 2 Gannan Medical University, Ganzhou, 341000 China; 3 Department of General Surgery, The First Affiliated Hospital of Nanchang University, Nanchang, 330000 China

**Keywords:** Robotic surgery, Natural orifice specimen extraction, Transvaginal specimen extraction, Sigmoid colon cancer, Rectal cancer

## Abstract

**Background:**

Colorectal cancer resection surgery with transvaginal specimen extraction is becoming increasingly accepted and used by surgeons. However, few publications on robotic anterior sigmoid colon and rectal cancer resection with transvaginal specimen extraction (TVSE) have been reported, and a clinical outcome comparison between conventional robotic minilaparotomy (LAP) and transvaginal specimen extraction in anterior sigmoid colon and rectal cancer resection has not been performed. The current study compared the short- and long-term outcomes of TVSE and LAP for sigmoid colon cancer and rectal cancer in a mono-institution.

**Methods:**

From December 2014 to October 2018, 45 patients who underwent TVSE and 45 patients who underwent LAP matched by tumor location, tumor stage, body mass index (BMI), American Society of Anesthesiologists (ASA) classification, gender, and age at the same period were included in the current study. The short- and long-term outcomes of TVSE and LAP were discussed.

**Results:**

No significant differences were found in patient characteristics. For the short-term outcomes, the operative time in the TVSE group was longer than that in the LAP group, and the postoperative pain and additional analgesia were lower in the TVSE group. Patients in the TVSE group required slightly less time to pass first flatus. There were no significant differences in overall complications, time to regular diet, length of hospital stay after surgery, estimated blood loss, or pathological outcomes. For long-term outcomes, the 3-year overall survival (94.9% vs. 91.7%, *p* = 0.702) and 3-year disease-free survival (88.4% vs. 86.2%, *p* = 0.758) were comparable between the two groups.

**Conclusion:**

The robotic TVSE is safe and feasible in selected sigmoid/upper rectal cancer patients with tumor diameter < 5 cm. This approach has slightly better short-term outcomes in terms of less postoperative pain and less analgesic requirements without any significant difference in long-term outcomes.

## Introduction

Colorectal cancer is the fifth most commonly diagnosed cancer and the fifth leading cause of cancer death. Nearly 3,763,000 people were diagnosed with colorectal cancer in China in 2015, and this number is estimated to increase annually according to the latest cancer statistics in China [1]. Surgery plays a major role in the treatment of colorectal cancer, and it is the cornerstone of curative treatment [2]. With the advent of robotic surgical systems, colorectal surgery experienced remarkable development over the past two decades; additionally, robotic surgical systems provide surgeons with better exposure and greater ergonomic comfort via superior dexterity and precise movement of the robotic arms during the dissection of small anatomical structures [3, 4]. Many studies and long-term follow-ups showed that the oncological outcomes of the robotic approach are comparable with those of laparoscopic surgery [5–7]. However, current robotic techniques require an abdominal incision for reconstruction of the digestive tract and removal of the surgical specimen. This additional incision leads to an additional risk of wound-related infection and incisional hernia [8, 9]. The natural orifice specimen extraction (NOSE) concept uses transanal or transvaginal routes instead of an additional incision for specimen extraction [10–12]. The transvaginal route is a valid option for the removal of colorectal specimens [12, 13]. Few studies have reported the safety and feasibility of transvaginal specimen removal in laparoscopic colorectal surgery [12–15]. Additionally, few publications have reported the clinical outcomes of robotic anterior resection with transvaginal NOSE for sigmoid colon cancer or rectal cancer, and no study has compared the short- and long-term outcomes with a conventional robotic procedure [16]. Transvaginal specimen extraction has been used in robotic anterior resection for sigmoid colon cancer and rectal cancer in our institution since 2014. Therefore, we performed the present study of 90 patients to compare the short- and long-term outcomes of TVSE and LAP for sigmoid colon cancer and rectal cancer.

## Methods

### Patients

We retrospectively analyzed the data of all patients who underwent robotic anterior resection for colorectal cancer from December 2014 to October 2018, at The First Affiliated Hospital of Nanchang University. Among 721 patients with colorectal cancer, 45 female patients who underwent TVSE were discovered. Forty-five patients who underwent LAP were matched individually using the following factors: tumor location, tumor stage, body mass index (BMI), American Society of Anesthesiologists (ASA) classification, gender, and age during the same period. We compared the short- and long-term outcomes between the two groups. The following inclusion and exclusion criteria were used.

Inclusion criteria: (1) Patients who were diagnosed with sigmoid or rectal cancer; (2) tumor size < 5 cm; (3) distance of tumor from the anal verge of 6–30 cm; (4) patients who gave approval for the corresponding approach; and (5) a preoperative evaluation that showed no local extensive infiltration or distant metastasis.

Exclusion criteria: (1) Patients who had contraindications for transvaginal surgical techniques, including virginity, pelvic anomaly, and pelvic inflammatory diseases; (2) child-bearing age; (3) patients who declined to give informed consent; (4) patients with missing clinical medical record information; and (5) patients who required diversion stoma.

These conditions were also applicable in the LAP group. All patients provided informed consent, and all of the procedures complied with the Declaration of Helsinki. The institutional review board of The First Affiliated Hospital of Nanchang University approved this retrospective study. Data on patient characteristics, short-term outcomes, and long-term outcomes were collected and compared between the two groups. The duration of the procedure did not include the docking time. Patient-controlled analgesia was used for all patients in the immediate postoperative period. Additional intravenous analgesia was administered when patients experienced prolonged pain in the ward. The largest size specified in the pathology reports was accepted as the tumor size. Pain scores were evaluated using a validated visual analog scale (VAS) that ranged from 0 to 10, with 0 representing no pain and 10 representing the worst conceivable pain. Pain was measured without analgesics on the morning of postoperative day 1. The patients were called on the telephone during the follow-up period. The clinical coordinator specifically asked the patients various questions about physical status and the results of regular review.

### Surgical techniques

**Fig. 1:**
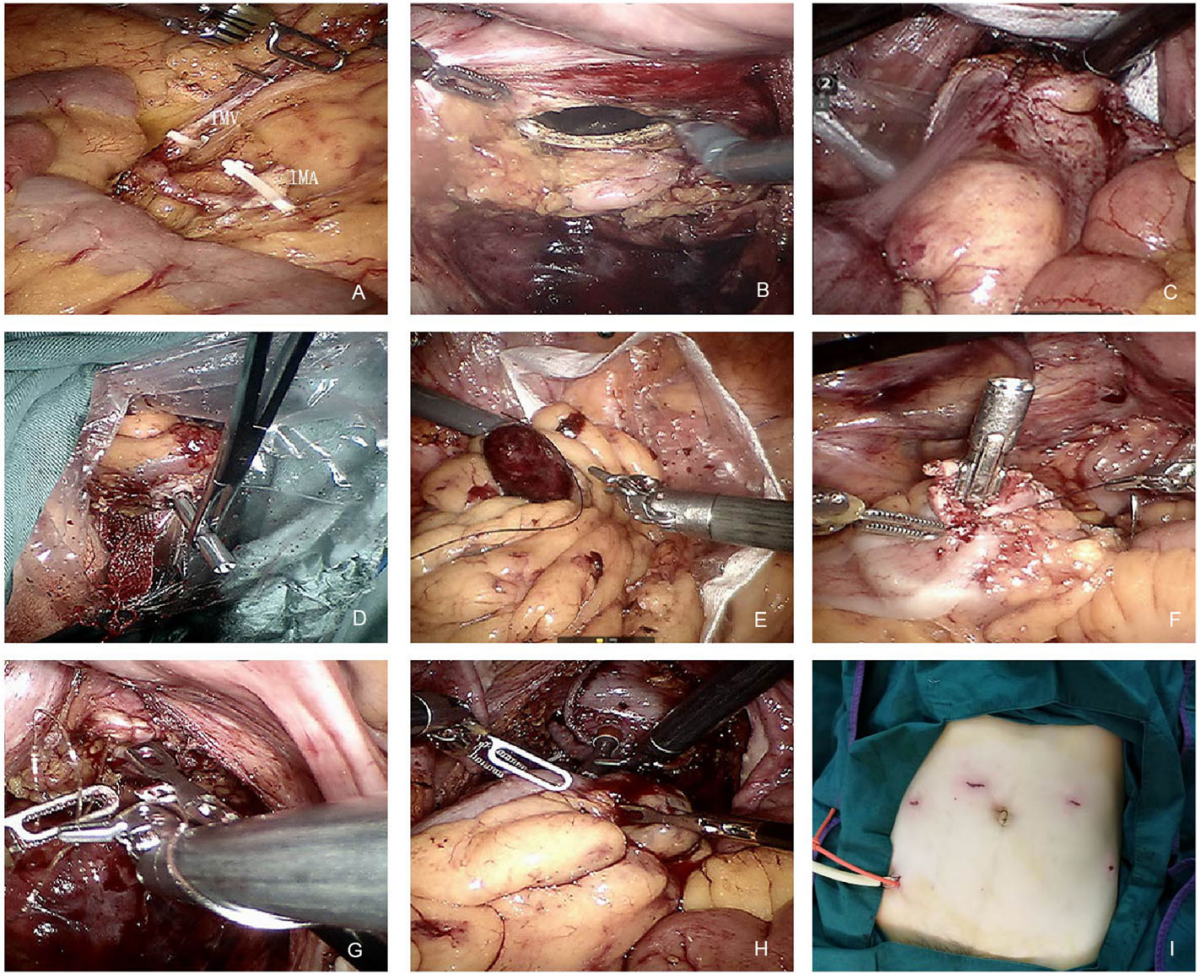
TVSE surgical procedure. **a** High tie of the inferior mesenteric artery and inferior mesenteric vein. **b** A 4­cm posterior colpotomy was performed. **c** Transvaginal exteriorization of the colon. **d** Endoluminal, proximal anastomotic site exteriorized. **e** Plastic bag with the specimen was removed through the vaginal incision. **f** Endoluminal, through a colpotomy above the diseased segment, proximal anastomotic site not exteriorized. **g** The posterior incisions were closed using a self­anchoring barbed suture. **h** Intracorporeal end-to-end colorectal anastomosis. **i** Postoperative abdominal wall without auxiliary incision

A specific surgeon (TYL) with extensive experience in robotic surgery performed all of the procedures. The Da Vinci Si and Da Vinci Xi Robot Surgical System (Intuitive Surgical, Sunnyvale, California, USA) were used for all procedures. All patients underwent endotracheal intubation and general anesthesia. During surgery, the patients were placed in a modified lithotomy, Trendelenburg position with a pneumoperitoneum of 12-15 mmHg. The single-docking technique with five ports was used for all patients, as described in previous studies [5]. The surgical technique was the same for TVSE and LAP except for the specimen extraction approach [17]. Following the division of the inferior mesenteric artery and full mobilization of the left colon, the rectum was mobilized using an avascular plan. The splenic flexure of the colon was not routinely mobilized but depended on the tension of the anastomosis. For rectal cancer, total mesorectal excision with a nerve-preserving technique was used in the mobilization of the rectum. For sigmoid colon cancer, the proximal and distal bowel was resected transversely approximately 10 cm from the tumor using a linear stapler. For rectal cancer, the distal rectum was resected at least 2 cm from the tumor using a linear stapler. The uterus was lifted by a robotic arm to obtain better visualization of the pelvis. After the vagina was washed using 10% povidone-iodine, a 4­cm posterior colpotomy was performed using a robotic harmonic knife. For rectal cancer, a sterile plastic sleeve was introduced via the vaginal incision, and transvaginal exteriorization of the colon, including the proximal site of anastomosis, and placement of the anvil directly at the level of the anastomosis, were performed. The proximal colon was gently re-introduced into the peritoneal cavity. For sigmoid colon cancer, a plastic bag was introduced through the assistant port after the posterior colpotomy was performed, and the specimen was placed inside the bag. The plastic bag with the specimen was removed through the vaginal incision under robotic guidance. The anvil of a circular stapler was introduced via the vaginal incision and placed at the end of the proximal colon using an intracorporeal purse-string suture. An intracorporeal end-to-end colorectal anastomosis was performed using a circular stapler. The colpotomy was sutured continuously with an absorbable suture using the robot. The surgical procedure in the TVSE group is shown in Fig. 1.

For conventional LAP, the same surgical procedures were used for colon and rectal mobilization. Rectal transection was accomplished intracorporeally using an endoscopic linear stapler via a 12-mm trocar on the right lower quadrant in all cases. The specimen was extracted via a 4- to 5-cm mini-laparotomy at the left lower quadrant trocar site using an incision protector. The bowel was anastomosed using the double-stapling method.

### Statistical analysis

Statistical calculations were performed using the IBM SPSS 24.0 software (SPSS Inc, IBM, Chicago, IL, USA). The mean ± standard deviation (SD) is used to present the descriptive variables. Student’s *t* test was used to analyze continuous data between the groups. Categorical variables were analyzed using Fisher’s exact test or chi-squared test. Survival curves were generated using the Kaplan–Meier method, and the differences in the survival curves were compared using the log-rank test. A *p* value < 0.05 was considered statistically significant.

## Results

### Clinical characteristics

Table 1 shows the baseline patient characteristics. No significant differences were detected between the TVSE and LAP groups in age, BMI, clinical tumor location, ASA score, tumor location, previous abdominal surgery, or postoperative chemotherapy.

**Table 1:** Patient characteristics

Characteristic	TVSE (*n* = 45)	LAP (*n* = 45)	*p* value
Age, year (mean ± SD)	58.1 ± 11.8	59.1 ± 10.8	0.683
BMI, kg/m^2^ (mean ± SD)	22.1 ± 2.7	21.6 ± 2.2	0.360
ASA score, *n* (%)			0.865
I	12 (26.7)	10 (22.2)	
II	28 (62.2)	29 (64.4)	
III	5 (11.1)	6 (13.4)	
Tumor location, *n* (%)			0.664
Sigmoid	16 (35.6)	18 (40)	
Rectum	29 (64.4)	27 (60)	
Previous abdominal surgery, *n* (%)			0.468
Presence	10 (22.2)	13 (28.9)	
Absence	35 (77.8)	32 (71.1)	
Clinical tumor category, *n* (%)			0.778
T1	10 (22.2)	9 (20.0)	
T2	12 (26.7)	15 (33.3)	
T3	23 (51.1)	21 (46.7)	
Postoperative chemotherapy, *n* (%)	9 (20.0)	10 (22.2)	0.796

*TVSE* totally robotic anterior resection with transvaginal specimen extraction, *LAP* robotic anterior resection with minilaparotomy, *SD* standard deviation, *BMI* body mass index, *ASA* American Society of Anesthesiologists

### Short-term outcomes

Table 2 shows the intraoperative and postoperative outcomes. Patients in both groups underwent similar surgeries. None of the patients required conversion to open surgery in either group. Intraoperative complications, which included bleeding and subcutaneous emphysema, occurred in 4 (8.8%) patients in the TVSE group and 3 (6.6%) patients in the LAP group. The TVSE group had a shorter time to first flatus (1.8 ± 0.7 vs. 2.1 ± 0.5 days, *p* = 0.045) and longer operation time (149.1 ± 26.3 vs. 131.8 ± 18.3 min, *p* < 0.001) than the LAP group. The postoperative pain on day 1 after surgery was lower in the TVSE group than the LAP group (pain score 4.1 ± 1.3 vs. 5.3 ± 1.6, *p* = 0.004). Fewer patients in the TVSE group required additional analgesia (3 (6.7%) vs. 12 (26.7%), *p* = 0.021). However, the time to regular diet, length of hospital stay after surgery, total hospital cost, and estimated blood loss showed no significant differences between the two groups. Four patients in the TVSE group and seven patients in the LAP group suffered postoperative complications. There were no cases of incisional hernia in either group. Two patients in the TVSE group developed pulmonary infections and were treated with antibiotics, and one patient developed an intra-abdominal abscess and received percutaneous drainage and antibiotics. One patient in the TVSE group developed anastomotic leakage, which was treated using a conservative treatment. Postoperative complications associated with the extraction site were not observed in the TVSE group. Two wound infections at the abdominal incision site occurred in the LAP group. Reoperation occurred in one patient who developed postoperative ileus in the LAP group. Other complications in the LAP group included one pneumonia, one intro-abdominal abscess, and two anastomotic leakage. No deaths occurred during the perioperative period in either group. No patients complained of pain at the site of the vaginal incision, and only 2 cases of vaginal spotting occurred after the surgery, which disappeared at the 4th and 6th weeks without treatment. According to the follow-up investigations, all patients resumed sexual activity, and none of them complained of difficulty in sexual intercourse.

**Table 2:** Intraoperative and postoperative outcomes

Parameters	TVSE (*n* = 45)	LAP (*n* = 45)	*p* value
Type of operation, *n* (%)			0.832
Anterior resection	25 (55.6)	26 (57.8)	
Low anterior resection	20 (44.4)	19 (42.2)	
Operation time, min (mean ± SD)	149.1 ± 26.3	131.8 ± 18.3	< 0.001
Estimated blood loss, mL (mean ± SD)	64.9.0 ± 36.5	79.5 ± 43.7	0.087
Intraoperative morbidity, *n* (%)	4 (8.8)	3 (6.6)	0.694
Pain score, VAS (mean ± SD)	4.1 ± 1.3	5.3 ± 1.6	0.004
Additional analgesics required, *n* (%)	3 (6.7)	12 (26.7)	0.021
Time to first flatus, day (mean ± SD)	1.8 ± 0.7	2.1 ± 0.5	0.045
Time to regular diet, day (mean ± SD)	2.1 ± 0.9	2.3 ± 1.1	0.280
Hospital stay after surgery, day (mean ± SD)	8.5 ± 2.8	9.8 ± 4.9	0.115
Total hospital cost, US dollars (mean ± SD)	9014.5	9448.6	0.159
Total complications, *n* (%)	4 (8.9)	7 (15.6)	0.522
Wound infection	0	2	
Pulmonary infection	2	1	
Intra-abdominal abscess	1	1	
Anastomotic leakage	1	2	
Ileus	0	1	

*TVSE* totally robotic anterior resection with transvaginal specimen extraction, *LAP* robotic anterior resection with minilaparotomy, *SD* standard deviation; *VAS* visual analogue scale

The pathological outcomes are described in Table 3. There were no significant differences in the TVSE group and LAP group in mean tumor size or the length of the resection margin. No patients had a positive resection margin. No significant difference in the mean number of retrieved lymph nodes was observed between the two groups. The histological differentiation and the tumor stages were similar between the two groups.

**Table 3:** Pathological characteristics

Parameters	TVSE (*n* = 45)	LAP (*n* = 45)	*p* value
Tumor size, cm (mean ± SD)	3.5 ± 1.3	3.6 ± 1.2	0.595
Proximal margin, cm (mean ± SD)	12.4 ± 8.6	11.6 ± 8.3	0.568
Distal margin, cm (mean ± SD)	3.8 ± 1.5	4.2 ± 2.3	0.326
Retrieved lymph node, *n* (mean ± SD)	15.9 ± 5.3	14.8 ± 4.7	0.360
Positive lymph node, n (mean ± SD)	1.1 ± 2.6	1.1 ± 2.7	1.000
Histologic differentiation, *n* (%)			0.950
Well	10 (22.2)	9 (20)	
Moderate	32 (71.1)	34 (75.6)	
Poor	3 (6.7)	2 (4.4)	
TNM stage, *n* (%)			0.857
I	10 (22.2)	12 (26.6)	
II	15 (33.4)	15 (33.4)	
III	20 (44.4)	18 (40)	

*TVSE* totally robotic anterior resection with transanal specimen extraction, *LAP* robotic anterior resection with minilaparotomy, *SD* standard deviation

### Long-term outcomes

**Fig. 2:**
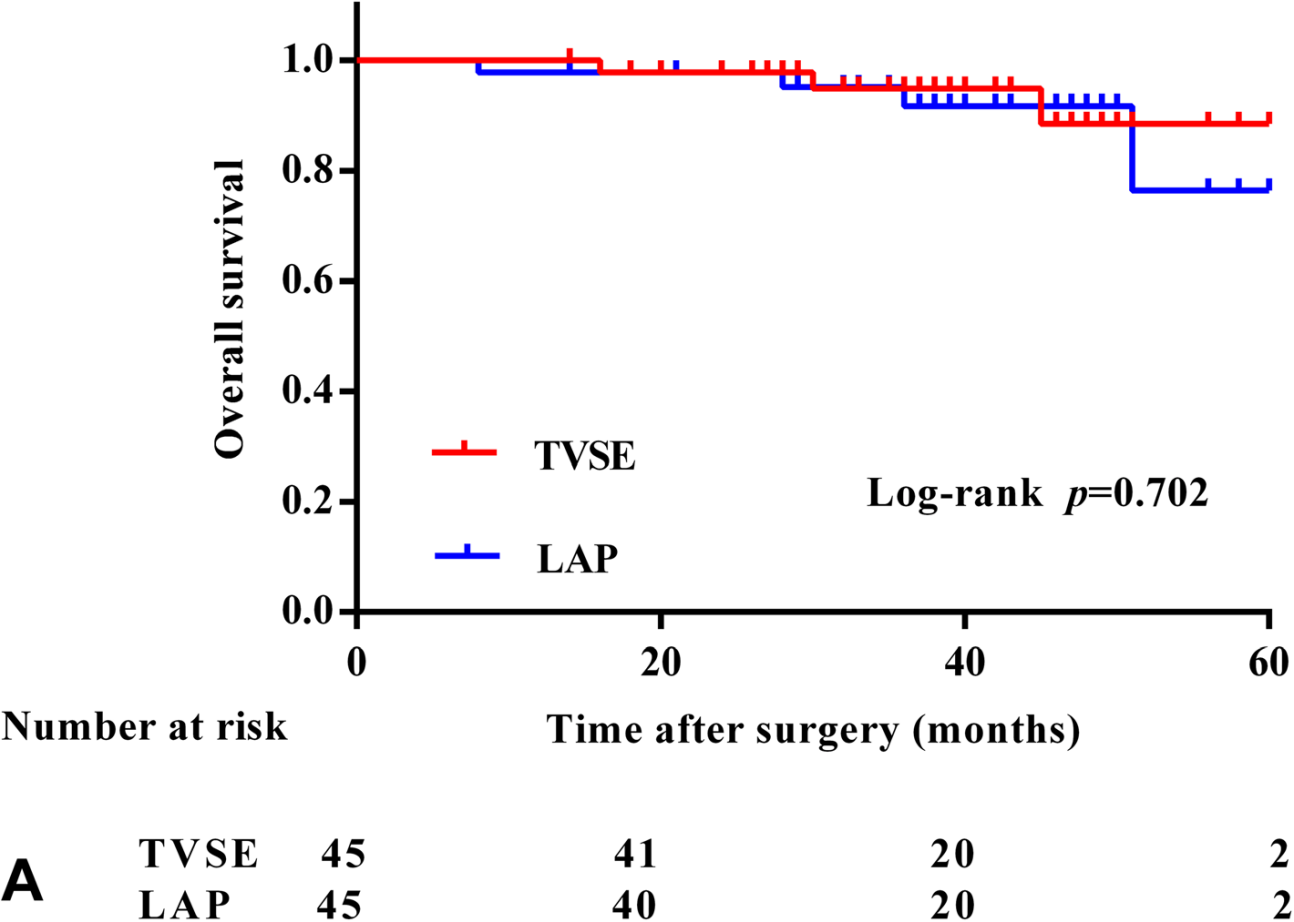
A 3-year overall survival (OS)

**Fig. 3:**
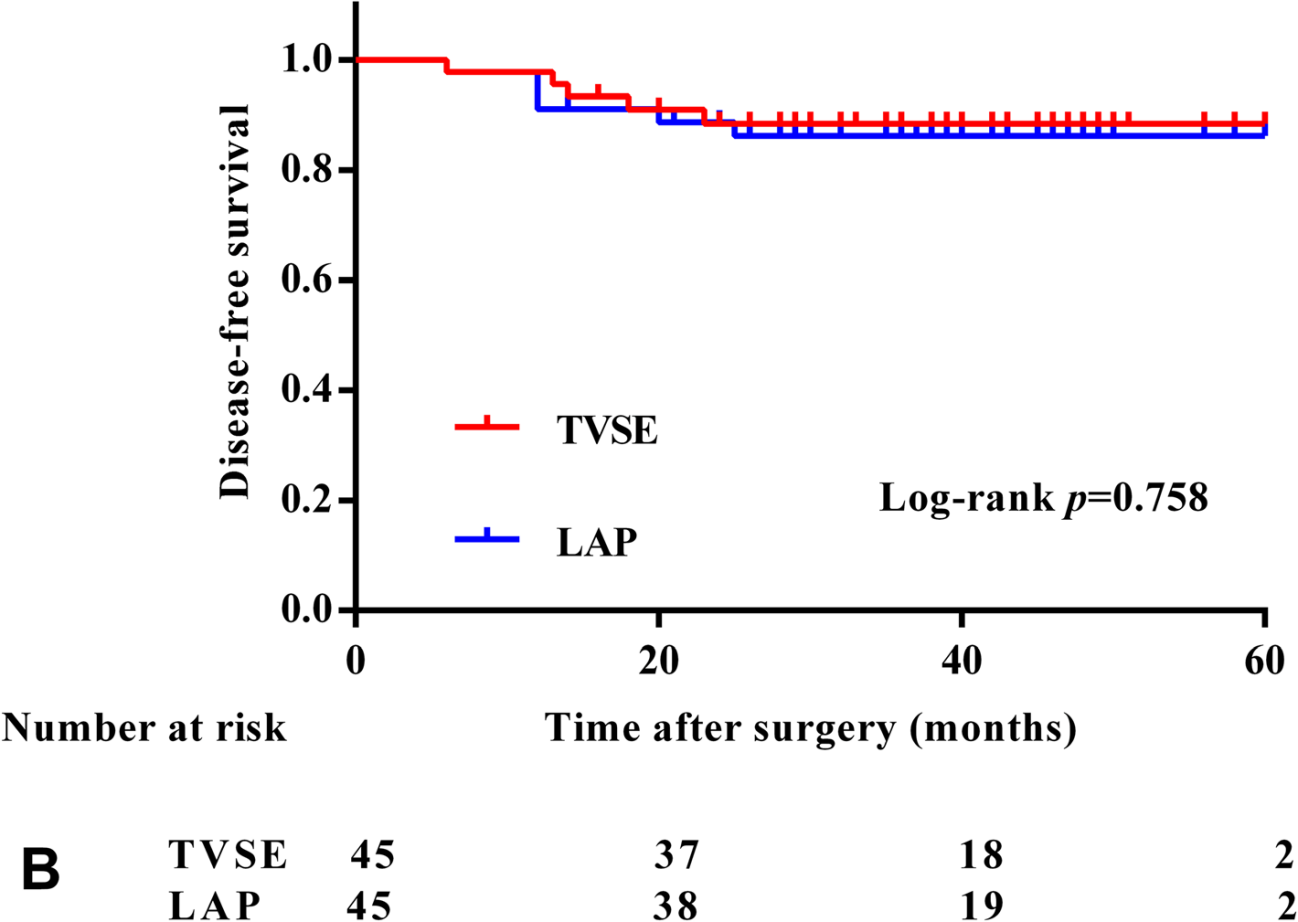
A 3-year disease-free survival (DFS)

The median follow-up time was 37.4 (range, 14–60) months. Seven of the 90 patients died, and 11 patients had a local recurrence or distant metastasis during the follow-up. Recurrences were observed in five patients in the TVSE group. One patient developed local recurrence, and four patients developed distant recurrences (three liver metastasis and one iliac bone metastasis). Four patients in the LAP group developed distant recurrence, and two patients developed local recurrence during the follow-up. Patients with local recurrence or distant metastasis received chemotherapy or surgery. Two patients in the TVSE group died at 37 months and 45 months due to hepatic metastasis, and one patient died at 27 months after bone metastasis. Three patients in the LAP group died at 8 months, 51 months, and 28 months after multiple hepatic recurrences, and one patient died at 8 months after peritoneal cavity metastasis. The 3-year overall survival rates were 94.9% and 91.7% in the TVSE and LAP groups (*p* = 0.702), respectively (Fig. 2). The 3-year disease-free survival rates were 88.4% and 86.2% in the TVSE and LAP groups (*p* = 0.758), respectively (Fig. 3).

## Discussion

Pigazzi first reported the use of robotic anterior resection for colorectal cancer resection in 2006 [18]. Since then, robotic surgery has been increasingly used in minimally invasive surgery centers worldwide. However, the additional incision in the abdominal wall for robotic anterior resection may influence or offset its minimal invasiveness in the treatment of colorectal cancer [8, 9]. The TVSE technique overcomes some of the drawbacks of robotic anterior resection, and it is widely used in colorectal surgery. However, there is scant information on transvaginal specimen extraction following robotic anterior resection for colorectal cancer. We performed this case-matched study to compare the short- and long- term outcomes between TVSE and LAP for sigmoid colon cancer and rectal cancer. Our results showed that the TVSE group experienced a longer operation time but an improved recovery of intestinal function, less postoperative pain, and lower analgesic requirements. The TVSE group achieved comparable surgical and oncological safety outcomes to the LAP group.

The operative time in the LAP group was significantly shorter than the TVSE group (131.8 ± 18.3 vs. 149.1 ± 26.3 min, *p* < 0.001), which is consistent with a previous retrospective study [12] and a meta-analysis [19]. The longer operative time of the TVSE group likely resulted from the time of opening and closing of the posterior fornix and the time needed for purse-string suturing. An assistant performed some procedures in the TVSE group, such as dilating, cleaning the vagina, inserting the specimen bag and the sterile plastic sleeve and removing the specimen. All of the procedures also significantly prolonged the operative time. Postoperative pain on day 1 after surgery was lower in the TVSE group than the LAP group. Several studies [10, 12, 20] showed similar results. This reduction may be explained by the fact that minilaparotomy, which traumatizes the abdominal wall, is more likely to cause vessel and nerve injury and lead to increased postoperative somatic pain at the incisional sites [21]. The posterior vaginal fornix is the lowest part of the pelvic cavity, and there are no large blood vessels or nerves in the area [22]. Therefore, patients in the TVSE group experienced less postoperative pain. The additional analgesia required was lower in the TVSE group than the LAP group (3 (6.7%) vs. 12 (26.7%), *p* = 0.021).

Previous studies [10, 12, 19] demonstrated that patients who underwent laparoscopic anterior resection with TVSE for colorectal cancer benefited from short-term outcomes, such as faster gastrointestinal recovery. The results of the current study also suggested that the TVSE group had a shorter time to first flatus. This result may be attributed to the fact that the TVSE technique is performed totally intraperitoneally, avoids gastrointestinal tract contact with the external air environment and minimizes stimulation in the abdominal cavity [23]. Patients in the TVSE group do not limit their ambulation due to the absence of incisional pain in the early postoperative period. Therefore, patients in the TVSE group experienced enhanced recovery of gastrointestinal function. However, the postoperative length of stay and total hospital cost did not differ between the TVSE group and the LAP group (*p* > 0.05). The similar total hospital cost between the two groups could be attributed to the fact that the surgical fee and consumables were almost identical. The length of hospital stay was longer in both study groups than reported in previous studies [12]. This difference may be attributed to the patients’ discharge willingness in our center, and a different health care delivery system, which makes the length of hospital stay heterogeneous [10]. An accelerated postoperative care plan was not used during this study, and patients had at least one bowel movement before the initiation of oral intake.

Postoperative complications are an essential factor for assessing the safety and feasibility of surgical procedures. Costantino FA et al. [24] performed a microbiological study of 22 patients and demonstrated that the contamination rate of peritoneal fluid was 100% in the NOSE group and 89% in the conventional laparoscopy group. However, the postoperative complications were not significantly different between the two groups. Similarly, there was no significant difference in the incidence of intra-abdominal abscesses between the two groups in our study. One possible reason for this result is the routine use of intraoperative peritoneal irrigation and intraoperative transvaginal lavage procedures in the TVSE group. For rectal cancer, the proximal colon was exteriorized through the vagina to place the anvil. Therefore, a half circumferential dichotomy or complete transection of the rectum was not required in the abdominal cavity. Severe anastomotic leakage may lead to failure of the entire surgery. The incidence of anastomotic leakage was similar in both groups in our study. Several studies [10, 19, 25] reported similar results. In addition to bacteriological concerns, major concerns after transvaginal NOSE in colorectal surgery are infertility and dyspareunia. However, a previous study [12] demonstrated that the effects of a transvaginal approach on postoperative vaginal sensation, pregnancy rate, and difficulty in sexual intercourse were not significant at the clinical level. Patients in the present study did not complain of colpotomy-related symptoms during the entire follow-up period, except for two patients who had vaginal spotting that recovered at the 4th and 6th weeks postoperatively without treatment. Therefore, we consider robotic anterior resection with transvaginal specimen extraction surgically safe.

The number of lymph nodes harvested and radical resection margins play an important role in the assessment of surgical quality. The intra-abdominal surgical procedures, including abdominal exploration, lymphadenectomy, and anastomosis, were almost identical in both groups, which indicates similar outcomes of retrieved lymph node and radical resection margin in both groups. No positive resection margins were observed in either group, and our study found that the average number of harvested lymph nodes was 15.9 in the TVSE group, which distinctly exceeded the recommended number that needs to be harvested in the 2017 National Comprehensive Cancer Network guidelines.

Few studies have compared long-term outcomes following TVSE and LAP for colorectal cancer. Kim et al. [12] conducted a case-matched study to compare the mid-term results between TVSE and LAP for colorectal cancer and found that 3-year disease-free survival rates were similar in the two groups. No port-site or transvaginal access-site recurrence events were observed during their follow-up. Similarly, a meta-analysis of 14 studies of 1435 patients concluded that laparoscopic colorectal surgery with NOSE surgery achieved comparable oncological safety to conventional laparoscopic surgery [19]. Transvaginal access-site recurrence events were not observed in the TVSE group in our study. One patient who developed local recurrence in the TVSE group had a 5-cm tumor and multiple regional lymph node metastases and refused to accept adjuvant chemotherapy due to poor physical condition after surgery. Therefore, the case of local recurrence may be associated with advanced primary tumors. Three-year overall survival and 3-year disease-free survival did not differ between the TVSE group and LAP group. The comparable oncological outcomes between the two groups may be attributed to the identical intra-abdominal procedures in both groups. The proper oncological principles and specimen handling were respected [17, 25]. Transvaginal specimen retrieval may not increase the complication rate, but only a skilled surgeon can perform this operation [26, 27].

There are some limitations of our study, including its retrospective nature and selection bias. To minimize these limitations, the preoperative data of patients in both groups were matched by tumor location, tumor stage, BMI, ASA, gender, and age. We excluded bulky tumors and advanced tumors to avoid the risk of damaging the vaginal wall and tumor seeding during specimen retrieval. Therefore, further studies are required to validate whether TVSE offers advantages to patients with bulky or advanced tumors. The sample size in this study was also relatively small. Further research, especially large prospective randomized studies, is necessary.

## Conclusions

The robotic TVSE is safe and feasible in selected sigmoid/upper rectal cancer patients with tumor diameter < 5 cm. This approach has slightly better short-term outcomes in terms of less postoperative pain and less analgesic requirements without any significant difference in long-term outcomes.

## Data Availability

Access to the database may be obtained from the corresponding author on reasonable request.

## Abbreviations

NOSE: Natural orifice specimen extraction
ASA: American Society of Anesthesiologists
BMI: body mass index
VAS: Visual analog scale
